# What “leaning in together” means to me

**DOI:** 10.1016/j.ijwd.2015.07.001

**Published:** 2015-09-26

**Authors:** Allison R. Larson

**Affiliations:** Department of Dermatology, Boston University School of Medicine, Boston Medical Center, Boston, MA

The Women’s Dermatologic Society luncheon at this year’s American Academy of Dermatology meeting featured a speaker whose talk centered on the problems associated with gender stereotypes in negotiations.Although much of her talk was focused on the impact this has on women, one of her final comments resonated with me: “Rigid ideas about gender roles are really limiting, especially for men.”I was so glad to hear her say this.For the last year, I have been wondering how many working professionals like me deal with this problem on a regular basis.Her words inspired me to write this article.

Let me take you back to one year ago.

It is time for annual reviews at my husband’s engineering company. He has received a mediocre rating once again. He patiently asks his boss about his co-worker, Ruth, who started at the same time he did, does similar work, and received a better rating this year. His boss explains that the work he is doing is fine, but that the goals his boss has set out for Ruth are different because she also has family responsibilities, and she has been able to meet her goals successfully. My husband doesn’t say anything—he doesn’t want to be seen as complaining, or worse, as being insensitive. When I hear about this that night, I am really surprised at what he was told.

“You should have told your boss you have to drop off and pick up our son every day, feed him dinner, and often put him to bed before I get home! He seems to think you have no family responsibilities. Doesn’t he know we have a kid?”

“Yes,” he tells me, “everyone knows.”

He makes sure to tell them every time he can’t rearrange his schedule to accommodate a last-minute late-afternoon meeting or when he is unable to put in the extra evening hours that others can but instead works with incredible efficiency in the hours he is there and always finishes his work ontime.

“They think I’m not interested in career advancement,” he tells me sadly.

Frustration builds inside me. He is a hardworking, ambitious person. All the people at his (now former) company seem to see is someone who has to leave by a certain time, and even when they know he is leaving to pick up our son, it still looks lazy. There are probably many workplaces out there that understand that a growing number of working men are assuming what I like to call the “primary caregiver role” in their families, but in the male-dominated tech sector that my husband works in, this still seems to be a novel idea. As a man who works full-time, my husband is expected to accommodate last-minute meetings. Unfortunately for him, his coworkers and bosses have had a hard time understanding that he cannot always do this because he has to incorporate childcare obligations into his workday plans. In our family, he has given me the flexibility to stay late at work when I need to and, in doing so, has sacrificed that flexibility for himself.

Last year, he and I went to see a local theater company perform *Rapture, Blister, Burn*, whose theme centered on women not being able to “have it all” with both a stellar career and fulfilling family life.It really resonated with both of us. We talked about how, more and more, the myth that women can “have everything” is being dispelled. I went on about the play for awhile, explaining how, as a working mom, I cannot be perfect both as an employee and as a parent, and how I need (and receive) a lot of support to be able to do both. My husband paused and then said quietly, “I wonder if there will come a time when people will write plays and books about this topic for men, too.”He reflected on some of the tough choices he has made in his job—to not work overtime like his colleagues, realizing it would hurt his ratings and chance for promotion. He knows our family relies on him to be there to take care of the kids at night. He secretly loves being a family man. He is great with our (now two) children, and he manages to cook dinner while taking care of them during a prime melt-down hour with ease. I know he is proud of that, but he does not talk about it much with his work buddies. I wonder what things would be like if men at his work stood around the water cooler bragging about the delicate balance of parenting while working full-time.

We as a society have made huge strides in the recognition that work–life balance is difficult for working moms. Support in the workplace has flourished in recent years. What about support for working dads? I sincerely believe that more men would be primary caregivers in their families if this was recognized as a valid path and supported as such. As I have seen first-hand, “rigid ideas about gender roles are really limiting, especially for men.”

I see some signs that we are moving closer to gender equality at work and at home. Sheryl Sandberg launched an initiative this year called “Lean In Together,” which focuses on encouraging men to take on a larger role at home. She sees that this is not just a “men’s issue,”but both a men’s and women’s issue. Once men and women are equally free to “lean in” at work and at home, we will have taken a large step toward equality. I also have seen the role of the stay-at-home dad receiving more positive feedback in recent years. I would like to see the working dad receive an equal amount of praise. My husband and I both refer to ourselves as working parents, but truthfully, outside of our house I have only ever heard the term “working mom” and never “working dad.”

My father-in-law recently asked me if I was “babysitting the kids” one afternoon and I burst out laughing. I kindly corrected this phrase to “parenting” and thanked him for making this mistake because I have heard the term “babysitting” used countless times when my husband takes care of our kids, but never before had it applied to me. I believe we are slowly moving toward equality. The more that working dads, spouses, and coworkers of working dads think and talk about work–life balance, the quicker our society will be able to eliminate these rigid gender roles. We will know we have succeeded in “leaning in together” when both men and women are free to choose their roles at work and at home and when their families, coworkers, and bosses are understanding and supportive of these choices.

